# Pyrolytic Transformation of Zn-TAL Metal–Organic Framework into Hollow Zn–N–C Spheres for Improved Oxygen Reduction Reaction Catalysis

**DOI:** 10.1021/acsomega.4c11318

**Published:** 2025-04-12

**Authors:** Gulnara Yusibova, John C. Douglin, Iuliia Vetik, Jekaterina Pozdnjakova, Kefeng Ping, Jaan Aruväli, Arvo Kikas, Vambola Kisand, Maike Käärik, Jaan Leis, Tiit Kaljuvee, Peeter Paaver, Sven Oras, Łukasz Ciupiński, Tomasz Plocinski, Marina Konuhova, Anatoli I. Popov, Dario R. Dekel, Vladislav Ivaništšev, Nadezda Kongi

**Affiliations:** † Institute of Chemistry, University of Tartu, Ravila 14a, 50411 Tartu, Estonia; ‡ The Wolfson Department of Chemical Engineering, Technion—Israel Institute of Technology, 3200003 Haifa, Israel; § Yichang Humanwell Pharmaceutical Co., Ltd, 19 Dalian Rd, Xiling District, Yichang, 443005 Hubei, China; ∥ Institute of Ecology and Earth Sciences, University of Tartu, Ravila 14a, 50411 Tartu, Estonia; ⊥ Institute of Physics, University of Tartu, Ostwaldi 1, 50411 Tartu, Estonia; # Department of Materials and Environmental Technology, Tallinn Technical University, Ehitajate tee 5, 19086 Tallinn, Estonia; ¶ Institute of Technology, University of Tartu, Nooruse 1, 50411 Tartu, Estonia; ∇ Faculty of Materials Science and Engineering, Warsaw University of Technology, Woloska 141, 02-507 Warsaw, Poland; ○ Institute of Solid State Physics, University of Latvia, 8 Kengaraga, LV-1063 Riga, Latvia; ⧫ The Nancy & Stephen Grand Technion Energy Program (GTEP), Technion—Israel Institute of Technology, 3200003 Haifa, Israel

## Abstract

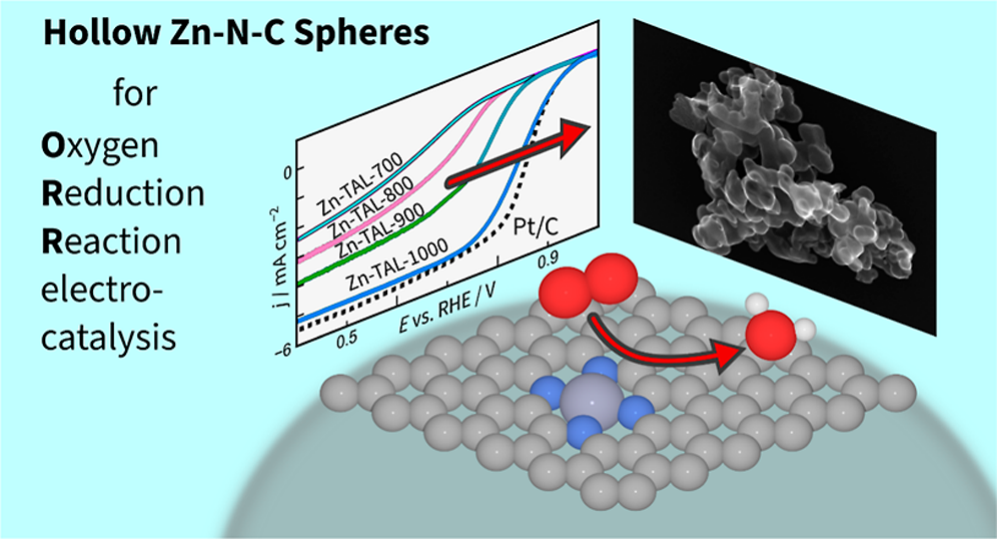

Metal–organic frameworks (MOFs) are promising precursors for creating metal–nitrogen–carbon (M–N–C) electrocatalysts with high performance, though maintaining their structure during pyrolysis is challenging. This study examines the transformation of a Zn-based MOF into an M–N–C electrocatalyst, focusing on the preservation of the carbon framework and the prevention of Zn aggregation during pyrolysis. A highly porous Zn–N–C electrocatalyst derived from Zn-TAL MOF (where TAL stands for the TalTech-UniTartu Alliance Laboratory) was synthesized via optimized pyrolysis, yielding notable electrocatalytic activity toward oxygen reduction reaction (ORR). Scanning electron microscopy (SEM) and X-ray diffraction spectroscopy (XRD) analyses confirmed that the carbon framework preserved its integrity and remained free of Zn metal aggregates, even at elevated temperatures. Rotating disc electrode (RDE) tests in an alkaline solution showed that the optimized Zn–N–C electrocatalyst demonstrated ORR activity on par with commercial Pt/C electrocatalysts. In an anion-exchange membrane fuel cell (AEMFC), the Zn–N–C material pyrolyzed at 1000 °C exhibited a peak power density of 553 mW cm^–2^ at 60 °C. This work demonstrates that Zn-TAL MOF is an excellent precursor for forming hollow Zn–N–C structures, making it a promising high-performance Pt-free electrocatalyst for fuel cells.

## 1 Introduction

The oxygen reduction reaction (ORR) has a critical role in next-generation renewable energy storage and conversion systems, including metal-air batteries and fuel cells.^1^ However, the ORR is kinetically sluggish, which poses a significant challenge to the practical usability of these technologies.^2,3^ To speed up the ORR process, novel cathode electrocatalyst materials are continuously being researched and developed.^4^ Despite these efforts, Pt-group metal-based electrocatalyst materials remain state-of-the-art due to their exceptional electrocatalytic activity.^5^ However, their high cost, scarcity, and poor stability significantly restrict their application at a large scale.^6^ In this context, transition metal–nitrogen–carbon (M–N–C) single-atom catalysts (SACs) emerge as highly promising and cost-effective alternatives, demonstrating exceptional efficiency in oxygen electrocatalysis.^7^ The nitrogen-coordinated metal sites (denoted as M–N_*x*_) integrated within the carbon framework of M–N–C materials are widely recognized as the primary catalytic sites for ORR.^8,9^ In addition to the M–N_*x*_ sites, the electrocatalytic behavior is significantly influenced by factors such as surface morphology and texture, and the diversity of nitrogen species, with the quantity of pyridinic nitrogen playing a particularly important role.^10−13^

The widespread method for preparing M–N–C electrocatalysts involves pyrolyzing a mixture of metal, nitrogen, and carbon-based precursors. Various precursors have been used to obtain highly dispersed active site catalysts. Among them, metal–organic framework (MOF) derived M–N–C electrocatalysts exhibit great potential due to their rich porosity, high surface area, and mechanical stability.^14−17^ Specifically, Zn-based M–N–C electrocatalysts exhibit intrinsic resistance to Fenton-like reactions, allowing them to retain robust durability in harsh environments.^18^ Tuning the organic ligands in MOF precursors allows control over the porosity of the resulting carbon materials.^19^ During heat treatment, the porous structure is maintained, which facilitates molecular transport and increases the dispersion of active sites. However, according to some studies, the structure of MOF-derived M–N–C materials changes after pyrolysis.^20−22^ Despite many research findings, the chemical and morphological transformations of MOFs during pyrolysis remain unclear because the structure of the derived product can only be studied after the synthesis process. Lately, various studies have been conducted to explore the relationship between the temperature of pyrolysis and the electrocatalytic behavior of materials.^23^ Ye et al. reported Zn-MOF-74 derived nitrogen-doped carbon pyrolyzed at 1000 °C which showed an ORR onset potential of 1.02 V and a half-wave potential (*E*_1/2_) of 0.90 V.^24^ Additionally, ZIF-8 has served as a template for synthesizing core–shell ZIF-8@ZIF-67 structures, leading to the formation of cobalt nanoparticles, as reported by Pan et al. and Liu et al.^25,26^

The uncertainty in morphological changes during pyrolysis also leads to challenges in understanding its effect on the electrochemical performance of catalysts. Several studies have investigated the use of Zn-MOF for the preparation of M–N–C electrocatalysts, revealing contradictory perspectives on the fate of zinc during the pyrolysis process. Some research suggests that zinc remains incorporated within the structure as Zn–N–C, contributing to the catalytic properties, while other studies indicate that zinc may evaporate during high-temperature treatment, potentially affecting the electrocatalytic performance.^27^ Li et al. conducted a systematic investigation using in situ analyses, such as in situ diffuse reflectance Fourier transform infrared spectroscopy and thermogravimetric-differential scanning calorimetry, to clarify the decomposition mechanism of Zn-MOF, complemented by X-ray and cyclic voltammetry methods for assessing structural and surface properties.^28^ They found that pyrolysis results in an amorphous carbon–ZnO composite characterized by a highly porous structure, with increased temperatures leading to broader pore size distributions and enhanced surface area and pore volume. In their computational study, Jin et al. examined four single-vacancy and seven double-vacancy Zn–N–C graphene electrocatalysts, all demonstrating promising stability.^29^ Their findings indicated that nitrogen doping effectively modulates electron transfer, making Zn–N–C a promising electrocatalyst for ORR with an overpotential of 0.45 V.^29^

In addition to pyrolyzed M–N–C materials, another class of ORR electrocatalysts involves conductive MOFs, such as Ni_3_(HITP)_2_ and M_3_(HHTP)_2_, which have shown promising intrinsic catalytic activity without requiring pyrolysis.^30−33^ These materials exhibit well-defined metal–ligand coordination environments and π-conjugated organic linkers to achieve electronic conductivity and redox activity.^31^ However, despite their well-structured catalytic sites, their practical implementation is often hindered by structural instability under electrochemical conditions, particularly in alkaline environments, where ligand degradation and dissolution of metal centers can occur. Additionally, while conductive MOFs facilitate O_2_ reduction through ligand-centered or metal-based redox mechanisms, their activity and stability often lag those of pyrolyzed carbonaceous catalysts. A study on Ni_3_(HITP)_2_, for example, reveals that while the framework exhibits notable ORR activity and electron delocalization, it undergoes gradual loss of performance over extended cycling.^32^ To address these challenges, MOF-derived M–N–C catalysts, which retain the porosity of the original MOF while acquiring enhanced stability through graphitization during pyrolysis, present a more viable alternative.

In this work, we present a Zn-MOF developed from an electron-rich 1H-benzo[*d*]imidazole-5,6-diol ligand, used here as a single precursor to design a highly efficient oxygen reduction electrocatalyst. This Zn-TAL framework (where TAL stands for the TalTech-UniTartu Alliance Laboratory) is rich in carbon and nitrogen, with zinc metal as the central component. Building on earlier TAL frameworks first synthesized in 2019 with iron,^34^ this Zn-based version represents an evolution in the design of the x-TAL series. Pyrolysis at 1000 °C transformed Zn-TAL into a highly active and porous M–N–C electrocatalyst with favorable morphology and optimal combination of active sites for ORR.

## 2 Materials and Methods

### 2.1 Fabrication of Zn-TAL-Based Electrocatalyst Materials

1H-benzo[*d*]imidazole-5,6-diol was synthesized following previously published protocol.^35−38^ 1H-benzo[*d*]imidazole-5,6-diol (7.79 g, 43.7 mmol, 1.0 equiv) was added into HBr (48%, 50 mL), and the mixture was left to stir at 120 °C. After 4 h, the mixture was cooled down to 0 °C, and the precipitate was collected and washed with petroleum ether to give the desired compound as a colorless solid (4.59 g, 30.6 mmol, 70%). (^1^H NMR (400 MHz, dimethyl sulfoxide (DMSO)) δ 9.75 (s, 2H), 9.25 (s, 1H), 7.12 (s, 2H). ^13^C NMR (100 MHz, DMSO) δ 146.4, 136.9, 123.7, 98.4.)

**Figure 1:**
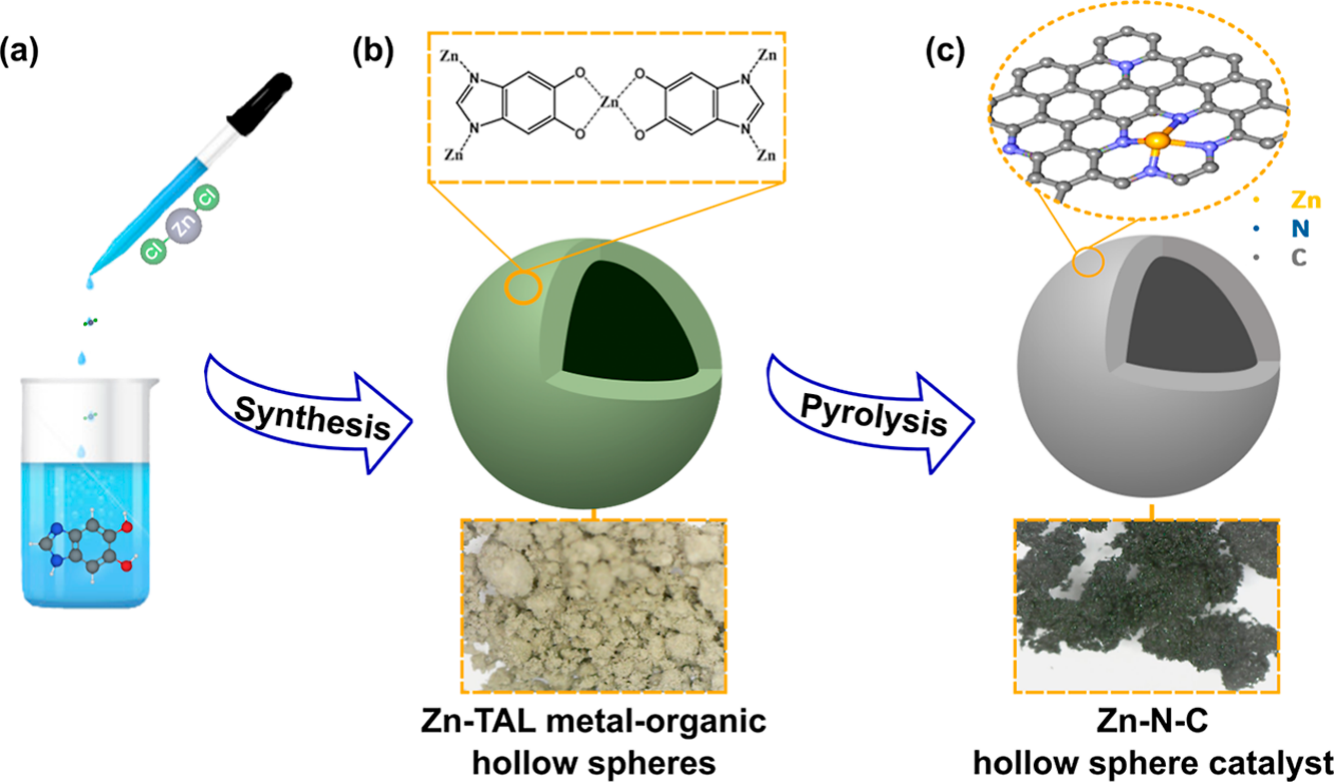
Schematic diagram of the (a) preparation of Zn-TAL; (b) schematic representation of a raw Zn-TAL hollow sphere; (c) schematic representation of a pyrolyzed hollow sphere.

The electrocatalyst synthesis strategy is illustrated in Figure 1. Zn-TAL MOF was synthesized by adding ZnCl_2_ (1.38 g, 10.1 mmol, 0.5 equiv) to a solution of 1H-benzo[*d*]imidazole-5,6-diol (3.0 g, 20.2 mmol, 2.0 equiv) in a solvent mixture of 25% aqueous NH_3_, dimethylformamide (DMF), EtOH, and water (4:10:10:15; 50 mL). The reaction mixture was stirred at room temperature for 24 h, after which the resulting solid was filtered, washed with ethanol, and dried at 60 °C for 12 h. The dried Zn-TAL was then subjected to pyrolysis under a nitrogen atmosphere for 1 h at four different temperatures (700, 800, 900, and 1000 °C) with a heating rate of 20 °C min^–1^. Following pyrolysis, the samples were acid-etched using 3 M HCl for 12 h at room temperature to remove residual zinc and create hollow, porous structures. However, acid treatment can leave behind Cl^–^ ions and other residual species within the carbon matrix, potentially affecting catalyst stability and performance.^39^ To eliminate these residues and further enhance material properties, the etched materials were subjected to a second pyrolysis step (repyrolysis). This step ensures complete acid removal and facilitates additional structural reorganization, improving conductivity, optimizing nitrogen coordination, and stabilizing active sites.^40,41^ The final Zn–N–C powders were labeled as Zn-TAL-700, Zn-TAL-800, Zn-TAL-900, and Zn-TAL-1000, corresponding to the respective pyrolysis temperatures.

### 2.2 Physical Characterization

As synthesized Zn-TAL was characterized by thermogravimetric analysis (TGA). The thermogravimetry differential thermal analysis (TG-DTA) was performed using a Setaram Labsys Evo 1600 thermal analyzer, samples were heated in an Ar atmosphere to 1000 °C at a heating rate of 10 °C min^–1^ under nonisothermal conditions. Standard alumina crucibles were used with a volume of 100 μL, while the sample mass was 50 mg, and the gas flow rate was set at 20 mL min^–1^.

The morphology of Zn-TAL samples was investigated by scanning electron microscope (SEM) with a cold field-emissions gun (CFEG) made by Hitachi High Technologies (Japan) model S5500, equipped with energy-dispersive X-ray spectrometer (EDX)—Thermo Fisher Noran System Six. The imaging was carried out at an accelerating voltage of 30 keV in secondary electron (SE) and bright field transmission (BF-STEM) mode at a magnification range of 10–200k times. The EDX analyses were performed at 5 and 30 keV. The lower energy was used to better characterize low energy peaks in the range of C, N, and Zn.

Powder X-ray diffraction (PXRD) studies were performed to gain information on the crystallography of the prepared samples using a Bruker D8 Advance diffractometer with Ni-filtered Cu Kα radiation. Surface elemental composition was investigated by X-ray photoelectron spectroscopy (XPS) employing Al Kα X-rays from a nonmonochromatic twin anode X-ray tube (Thermo XR3E2) and an electron energy analyzer SCIENTA SES 100. The metal content in studied materials was investigated by the microwave plasma atomic emission spectroscopy (MP-AES) technique. Analytical samples underwent preparation through digestion in the Anton Paar Multiwave PRO microwave system, utilizing NXF100 vessels (with PTFE/TFM liner) within an 8NXF100 rotor. Following digestion, the samples were diluted in 2% HNO_3_ to achieve a final dilution factor of 61,000 and subsequently analyzed using the Agilent 4210 MP-AES. Elemental analyses were conducted using the PerkinElmer@2400 Series II CHNSO/O Elemental Analyzer. The textural properties of the electrocatalysts were examined through low-temperature nitrogen adsorption conducted at the boiling point of nitrogen (77 K) using the NOVAtouch LX2 instrument from Quantachrome Instruments. Prior to measurement, the materials were degassed under a vacuum for 12 h at 300 °C. The BET surface area (*S*_BET_) of the samples was then determined within a *P*/*P*_0_ range of 0.02–0.2. The overall pore volume (*V*_tot_) was assessed at *P*/*P*_0_ 0.97. Pore size distribution (PSD) and specific surface area (*S*_dft_) were derived from N_2_ isotherms employing a quenched solid density functional theory (QSDFT) equilibrium model designed for slit-type pores.

### 2.3 Electrochemical Characterization

Electrochemical measurements were conducted using a standard three-electrode configuration, with a glassy carbon (GC) disk electrode as the working electrode, a reversible hydrogen electrode (RHE) as the reference electrode, and a GC rod as the counter electrode. Autolab PGSTAT128N potentiostat/galvanostat controlled by Nova 2.1.7 software was used to apply the potential. A GC disc electrode (rotating disc electrode—RDE) was connected to the OrigaBox speed controller unit and rotated at various speeds (ω = 400, 620, 900, 1225, 1600, and 2025 rpm). Fresh alkaline electrolyte solutions were prepared by dissolving KOH pellets (99.99%, Sigma-Aldrich) in Milli-Q water. Electrolytes were saturated with pure O_2_ (99.999%, Linde Gas) for ORR experiments and with Ar (99.999%, Linde Gas) to eliminate oxygen for recording the cyclic voltammetry (CV) curves. The GC electrodes (diameter: 5 mm) were polished with 1 and 0.3 μm alumina slurries and sonicated in both isopropanol and Milli-Q water for 3 min to eliminate any remaining abrasive particles. 5 mg of electrocatalyst powder was dispersed in 200 μL of a 0.5% Nafion solution (Sigma-Aldrich) in 2-propanol and sonicated for 5 min to prepare a uniform ink. Then, 4 μL of the resulting electrocatalyst suspension was drop-cast onto a GC electrode with a mass loading of 0.5 mg cm^–2^ and dried in ambient air at room temperature. The commercial 20 wt % Pt/C (E-TEK) was employed as a benchmark for ORR using the same protocol for preparing ink and working electrodes. Selected electrocatalysts underwent accelerated stability testing according to a protocol of 5000 CV potential cycles from 1.0 to 0.6 V versus RHE, with a scan rate of 50 mV s^–1^, at a rotation rate of 1600 rpm in an O_2_-saturated electrolyte.

### 2.4 Anion Exchange Membrane Fuel Cell Testing

In order to illustrate the practical application in an anion exchange membrane fuel cell (AEMFC), the Zn-TAL-100 electrocatalyst was prepared as a cathode following procedures similar to our prior publications.^42−51^ The cathode and anode were loaded to 1 mg_Zn/TAL-1000_ cm^–2^ and 0.6 mgPtRu cm^–2^, respectively, while a high-density polyethylene (HDPE) based AEM was utilized. The AEMFC was tested in a Scribner Associates 850E test station operated at a cell temperature of 60 °C, with an cathode humidifier temperature set at 564 °C and a anode humidifier temperature at 54 °C. The gas flow rates for both oxygen and hydrogen were maintained at 1 standard liter per minute (SLPM), with a back-pressure of 100 kPag. The polarization curve was obtained by scanning from open-circuit voltage (OCV) of ∼1–0.1 V at a scan rate of 10 mV s^–1^.

## 3 Results and Discussion

### 3.1 Physicochemical Characterization

The selection of an appropriate ligand is critical for the design of catalyst precursors, as the ligand plays a crucial role in influencing the catalytic activity, selectivity, and stability of the resulting catalyst.^18,53^ In this study, 1H-benzo[*d*]imidazole-5,6-diol was chosen for its carbon and nitrogen-rich composition and its additional functional groups, which enhance its multidirectional ligating capabilities. Before pyrolysis, the structure of the Zn-TAL raw consisted of well-defined hollow, porous particles, as shown in the SEM images (Figure S1a, Supporting Information).

To better understand Zn-TAL behavior during pyrolysis, thermogravimetric analysis was conducted under conditions that mimic the pyrolysis process (Figure S1b). The TGA results revealed an initial weight loss between 0 and 160 °C, corresponding to the evaporation of water and residual solvents. As the temperature increased further, additional weight loss was observed between 160 and 600 °C, which is attributed to the decomposition of the organic components of the Zn-TAL. Around 650 °C, the weight loss was associated with the evaporation of clustered Zn particles, consistent with previous findings.^27^ The mass of the Zn-TAL sample continued to decline beyond 650 °C, without stabilizing at any plateau, indicating ongoing thermal decomposition.

**Figure 2:**
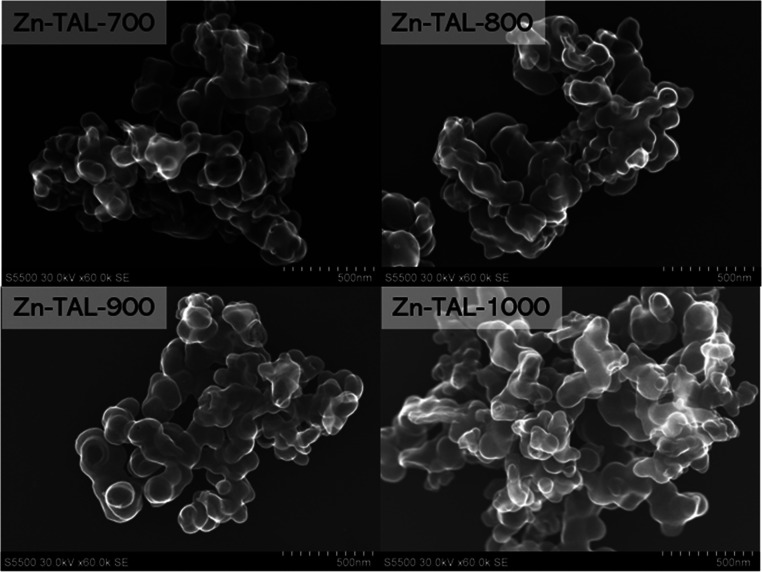
Scanning electron microscopy images (secondary electrons imaging mode) of Zn-TAL samples pyrolyzed at different temperatures (700, 800, 900, 1000 °C).

SEM studies confirmed that the nonpyrolyzed Zn-TAL raw exhibited the largest hollow spheres, averaging 140–160 nm in diameter, as shown in Figure S1a. As the temperature increases, the hollow spheres in Zn-MOF-700 shrink slightly to approximately 100–120 nm (Figure 2). Continued heating results in a further size reduction, with hollow spheres measuring between 60 and 100 nm. Notably, even at elevated temperatures, the carbon framework maintains its structural integrity. The only changes observed are in the dimensions of the hollow spheres, which remain free of visible Zn metal aggregates, which is consistent with the PXRD findings.

The corresponding energy dispersive spectroscopy (EDS) mapping results (Tables S1 and S2) clearly demonstrate the presence of zinc, oxygen, nitrogen and carbon elements on the surface of Zn-TAL materials. As the pyrolysis temperature increased, the surface content of Zn diminished significantly, decreasing from 3.87 at. % in the nonpyrolyzed Zn-TAL raw to 0.20 at. % in the sample treated at 1000 °C. This trend aligns with findings from the literature, which indicate that during high-temperature pyrolysis, Zn-based MOFs can convert to carbon materials with minimal residual metal due to the relatively low evaporation temperature of Zn. Correspondingly, the carbon content increased from 71.44 at. % in the nonpyrolyzed state to 94.65 at. % after pyrolysis at 1000 °C.

**Figure 3:**
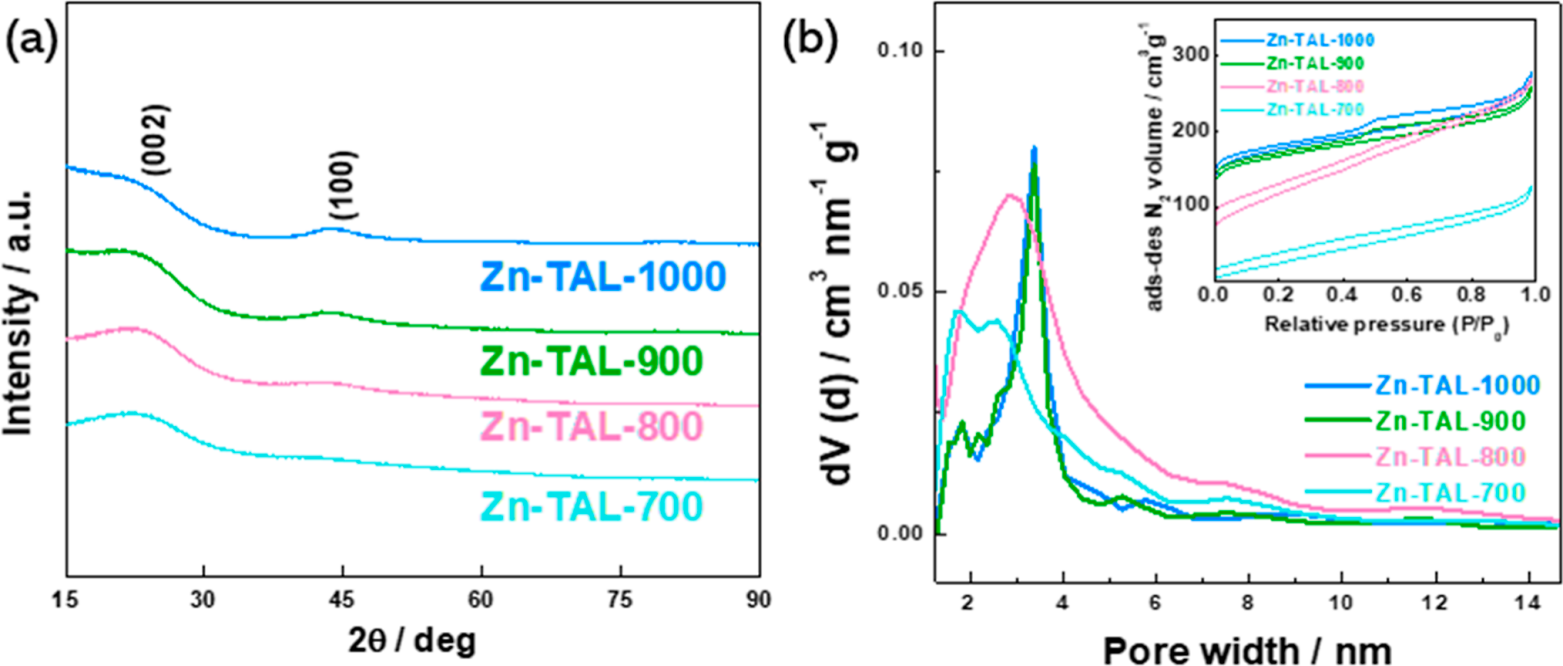
(a) X-ray diffraction patterns, (b) pore size distribution, and (inset) N_2_ sorption isotherms of Zn-TAL-700, Zn-TAL-800, Zn-TAL-900, and Zn-TAL-1000.

X-ray diffraction (XRD) analysis was conducted to investigate the composition and crystallographic structure of the electrocatalyst materials. The XRD patterns (Figure 3a) confirmed that all four pyrolyzed samples predominantly consisted of amorphous carbon, with no detectable peaks corresponding to residual Zn compounds. Despite this, some Zn likely remains in the materials, presumably coordinated with nitrogen in an atomically dispersed form. The peaks observed at 26.2° and 44.2° are attributed to the diffraction of the (002) and (100) planes of the graphite phase (PDF 01-077-7164).^54,55^ The carbon peak positions remained consistent across all samples, while their intensities systematically varied with temperature. As the pyrolysis temperature increased, the intensity of the (100) peaks slightly increased, indicating improved crystallinity and structural ordering, consistent with previous studies. In contrast, the intensity of the (002) peaks decreased with rising temperatures, suggesting a reduction in crystallite size or a possible phase transformation within the carbon structure due to thermal treatment.

To evaluate the specific surface area and pore size distribution of the synthesized electrocatalysts, N_2_ physisorption analysis was performed (Figure 3b). All samples exhibited typical Type-IV isotherms, accompanied by a Type H4 hysteresis loop in the relative pressure range of *P*/*P*_0_ > 0.4, indicating the coexistence of micro- and mesopores (inset to Figure 3b).^56^ The isotherms demonstrated a rapid increase at low relative pressures with increasing pyrolysis temperatures. Further analysis of the pore size distribution curves confirmed that the electrocatalysts were primarily composed of micropores and mesopores, with most mesopores having diameters ranging from 3 to 3.5 nm. Zinc serves as a morphology-controlling agent during pyrolysis,^57,58^ and as the temperature increases, the specific surface area of the material significantly enhances from 100 to 746 m^2^ g^–1^. This increase in porosity results in a higher proportion of electrochemically active sites, which further improves the ORR activity of the electrocatalysts. Key parameters, including specific surface area (*S*_DFT_), micropore volume (*V*_μ_), and total pore volume (*V*_tot_) for the Zn-TAL-derived electrocatalysts, were calculated and are summarized in Table 1.

**Table 1:** Textural Properties of Zn-TAL-Based Zn–N–C Materials

electrocatalyst	*S*_BET_ (m^2^ g^–1^)	*S*_DFT_ (m^2^ g^–1^)	*V*_tot_ (cm^3^ g^–1^)	*V*_μ_ (cm^3^ g^–1^)
Zn-TAL-700	103	100	0.17	0.03
Zn-TAL-800	414	475	0.40	0.14
Zn-TAL-900	584	715	0.37	0.23
Zn-TAL-1000	615	746	0.40	0.24

**Figure 4:**
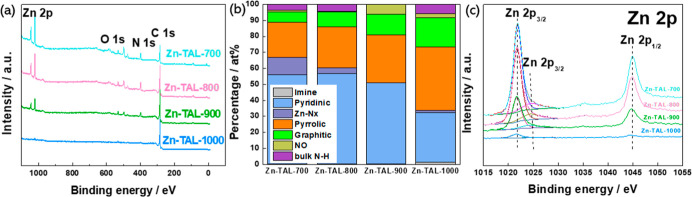
(a) The XPS survey spectra obtained for Zn-TAL derived electrocatalysts; (b) the bar plot of the different types of nitrogen species and their atomic weight percentage; (c) Deconvoluted Zn 2p XPS spectra for Zn-TAL derived electrocatalysts.

X-ray photoelectron spectroscopy was conducted to investigate the surface elemental composition of the synthesized electrocatalysts. The XPS survey spectra (Figure 4a) confirmed the presence of carbon, nitrogen, oxygen, and Zn in all the electrocatalysts, indicating that Zn was not fully evaporated during pyrolysis. The surface atomic concentrations of the elements are summarized in Table 2. As the pyrolysis temperature increased from 700 to 1000 °C, the Zn content decreased significantly from 9.53 to 0.44 at. %, suggesting progressive evaporation of Zn at higher temperatures. Additionally, chlorine residues from the synthesis process were detected in the samples pyrolyzed at 700 and 800 °C, but were completely removed after pyrolysis at 900 and 1000 °C. The oxygen and nitrogen content decreased progressively during pyrolysis. Meanwhile, the carbon content displayed a clear upward trend with increasing pyrolysis temperatures, reflecting a growing abundance of carbon-containing species. In the Zn-TAL-1000 sample, the surface contained the highest proportion of carbon (94.37 at. %), suggesting a higher degree of graphitization, which may enhance the stability of the electrocatalyst. The deconvoluted high-resolution XPS spectra in the C 1s region (Figure S2, Table S3) revealed various carbon species, including C–C (283.5 eV), C–O (285.8 eV), C=O (290 eV, 288 eV), C=C (283.7 eV), C–C/C=C, carbide (282.6 eV), and π–π* (291.5 eV).^59^ C–C and C=C species indicate the presence of graphitic and sp^2^-hybridized carbon structures, which contribute to enhanced electrical conductivity and structural stability. The increased graphitization at higher pyrolysis temperatures improves electron transport.

**Table 2:** Surface Elemental Composition of Zn-TAL-Derived Electrocatalysts (at. %) Obtained From XPS Analysis

electrocatalyst	C	N	O	Cl	Zn
Zn-TAL-700	68.52	15.39	5.3	1.25	9.53
Zn-TAL-800	76.66	12.82	4.42	0.42	5.68
Zn-TAL-900	85.54	8.38	3.53	0	2.55
Zn-TAL-1000	94.37	2.96	2.22	0	0.44

The deconvolution of high-resolution XPS spectra in the N 1s region revealed multiple nitrogen species, including pyridinic (398.3 eV), pyrrolic (400.6 eV), graphitic (401.8 eV), oxidized (403.7 eV), and metal-coordinated nitrogen (M–N_*x*_) groups (399.6 eV) as summarized in Figures 4b and S4. At higher pyrolysis temperatures, the structural and chemical composition of the carbon matrix undergoes significant transformation, as confirmed by both XRD and XPS analyses. The observed increase in the (100) peak intensity in XRD suggests improved structural ordering, indicative of graphitization. This is further supported by XPS data, which reveals a concurrent rise in graphitic nitrogen content. The incorporation of nitrogen into the carbon lattice in a graphitic configuration enhances electronic conductivity and structural stability, contributing to increased crystallinity. Additionally, the presence of pyrrolic nitrogen at elevated temperatures suggests the retention of edge defects and functionalities, which may facilitate active site exposure for catalytic applications. The decrease in the (002) peak intensity, often associated with layer stacking disruptions, aligns with the nitrogen doping effect, which can introduce disorder while simultaneously promoting a more open and accessible carbon framework.

A peak at 398.95 eV, corresponding to M–N_*x*_ species, was observed only in samples pyrolyzed at 700, 800, and 1000 °C. Notably, the surface concentration of M–N_*x*_ species decreased significantly from 1.7% at lower temperatures to just 0.04% at 1000 °C. At 700 and 800 °C, pyridinic nitrogen was the dominant species, making up about 56% of the total nitrogen content. However, after pyrolysis at 1000 °C, the proportion of pyridinic nitrogen dropped to 31%, while pyrrolic nitrogen increased from 22 to 39%. Mostly pyridinic, and M–N_*x*_ moieties are well-recognized for their role as highly active sites for ORR, contributing directly to the enhanced catalytic performance.^60^ Furthermore, it has been reported that pyridinic and graphitic nitrogen account for approximately 50% and 30%, respectively, of the active nitrogen sites involved in the ORR.^52,61^

Interestingly, for the Zn-TAL-900 sample, no apparent Zn–N_*x*_ species were observed despite the presence of 2.55 at. % Zn. One possible explanation is that at 900 °C, Zn atoms may be primarily present as ZnO or Zn oxynitride species (Zn–O–N) rather than Zn–N_*x*_. This is supported by the highest content of NO-related nitrogen species observed in the deconvoluted XPS spectra for Zn-TAL-900. Given that Zn can interact with oxygen even under nominally inert conditions due to residual oxygen or defects in the carbon matrix, it is plausible that Zn becomes incorporated into Zn–O species instead of forming Zn–N_*x*_ coordination. Furthermore, 900 °C may represent a transition temperature where Zn–N_*x*_ sites begin to destabilize, leading to the formation of more oxidized Zn species before significant Zn evaporation occurs at 1000 °C. At this stage, Zn may be trapped in oxide-rich domains or weakly bound to the carbon framework, making Zn–N_*x*_ coordination less detectable in XPS deconvolution.

Additionally, the distinctive peaks in high-resolution Zn 2p spectra, appearing at 1021.9 for 2p_3/2_ and at 1045 eV for 2p_1/2_ electronic configurations of Zn atoms, exhibit a characteristic spin–orbit splitting of approximately 23 eV (Figure 4c). As the pyrolysis temperature rises, the surface atomic concentration of Zn 2p species decreases, confirming the evaporation of metal species. This trend aligns with the bulk Zn content measured by microwave plasma-atomic emission spectroscopy (MP-AES), which shows that Zn-TAL-1000 has the lowest Zn concentration (0.23 wt %) compared to Zn-TAL-700, Zn-TAL-800, and Zn-TAL-900, which contain 1.43, 1.10, and 0.58 wt % of Zn, respectively. The decline in Zn content with increasing pyrolysis temperature is consistently observed across EDS, XPS, and MP-AES analyses, each reflecting different detection depths. EDS and XPS confirm significant Zn evaporation, while MP-AES further supports this trend, showing the lowest Zn concentration in the highest-temperature sample.

### 3.2 Electrochemical Characterization

**Figure 5:**
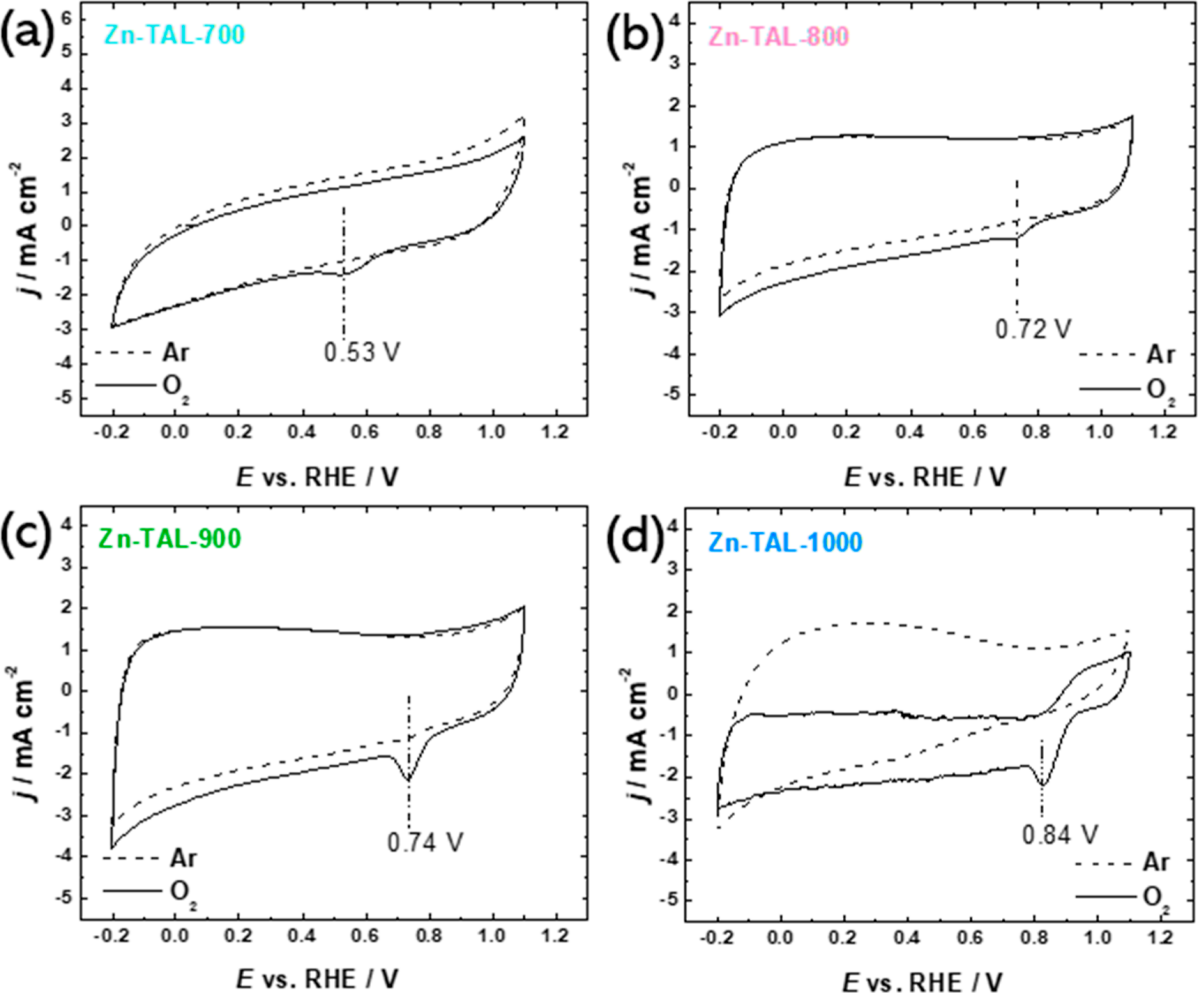
CV curves recorded for (a) Zn-TAL-700, (b) Zn-TAL-800, (c) Zn-TAL-900, and (d) Zn-TAL-1000 in Ar- (dashed line) and O_2_-saturated (solid line) 0.1 M KOH at 10 mV s^–1^.

**Figure 6:**
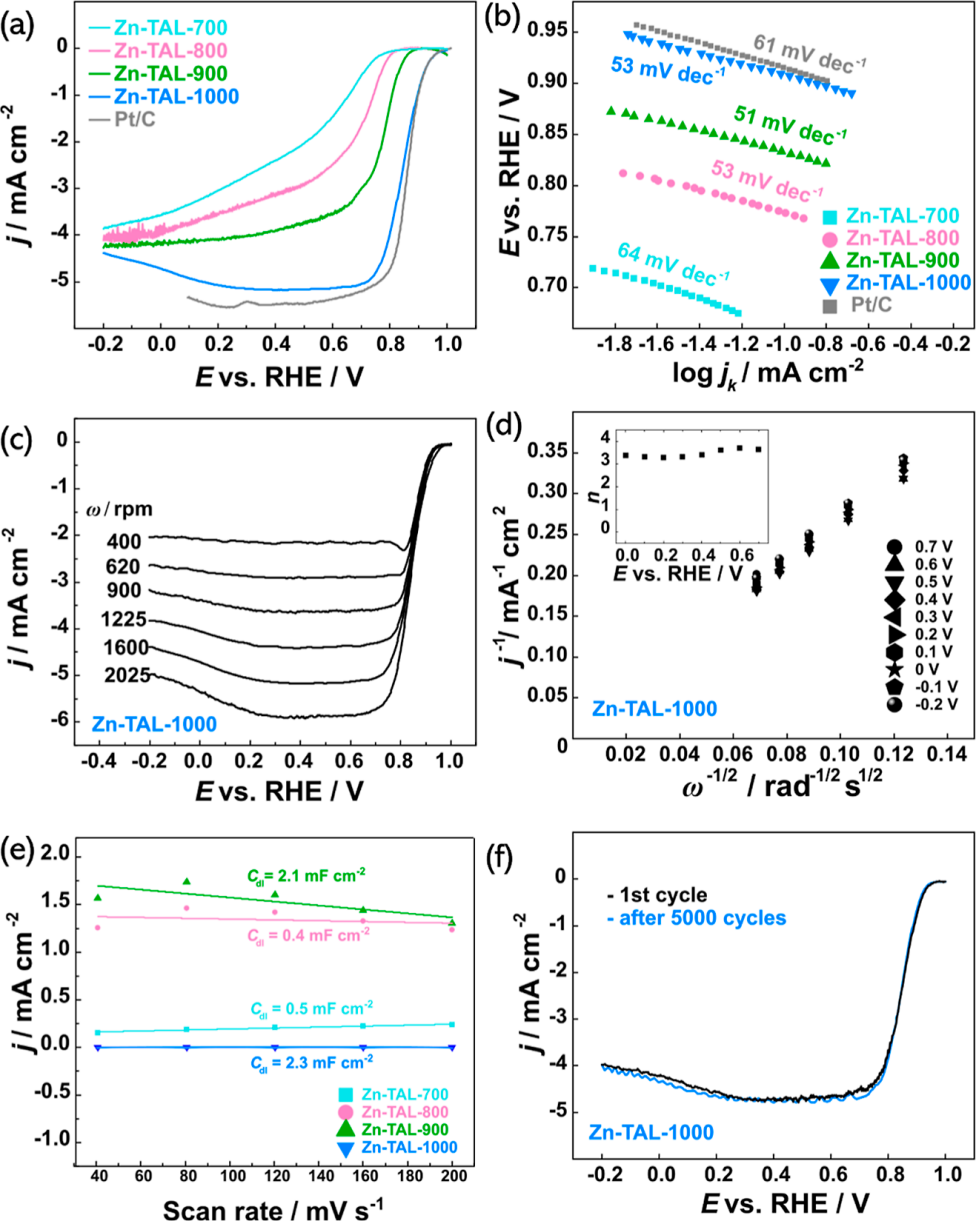
(a) Comparison of ORR polarization curves recorded for all Zn-TAL-derived samples and commercial Pt/C in O_2_-saturated 0.1 M KOH at 1600 rpm; (b) ORR Tafel plots derived from the RDE data; (c) ORR polarization curves recorded for Zn-TAL-1000 at different electrode rotation rates; (d) Koutecky–Levich plots constructed from RDE data on Zn-TAL-1000 (inset: number of electrons transferred (*n*)); (e) the charging current densities plotted against the scan rates for all studied Zn-TAL samples; (f) ORR polarization curves recorded for Zn-TAL-100 before and after 5000 cycles from 0.6 to 1.0 V vs RHE in O_2_-saturated KOH, 10 mV s^–1^, ω = 1600 rpm.

The ORR electrocatalytic behavior of all prepared electrocatalysts was studied and compared with commercial Pt/C. First, cyclic voltammetry experiments were conducted in 0.1 M KOH solution with either Ar or O_2_ saturation at a scan rate of 10 mV s^–1^. As seen from Figure 5, all four electrocatalysts showed distinguishable reduction current peaks in an O_2_-saturated electrolyte. As the pyrolysis temperature increased from 700 to 1000 °C, the reduction peak progressively shifted to 0.84 V vs RHE, indicating an enhancement in electrocatalytic performance with increasing pyrolysis temperature. To gain a deeper understanding of the specific activity of the prepared catalysts, their electrochemical active surface area (ECSA) was evaluated. This was achieved by cycling the samples in an Ar-saturated electrolyte at scan rates of 40, 80, 120, 160, and 200 mV s^–1^ to measure the electrochemical double-layer capacitance (*C*_dl_) (Figure S5). Cathodic and anodic current densities were recorded at non-Faradaic potentials, specifically within the range of 0.92–1.00 V vs RHE. The extracted *C*_dl_ values were plotted against the corresponding scan rates (Figure 6e), with the slope of the fitted trendline representing the *C*_dl_. The ECSA of the electrocatalysts was calculated using the equation
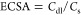
where *C*_s_ is the specific capacitance of the electrocatalyst. For the all TAL-derived materials, a *C*_s_ value of 0.040 mF cm^–2^ was used, based on reported values.^62−64^ Among the synthesized materials, Zn-TAL-1000 exhibited the highest ECSA (57.5 cm^2^) followed by Zn-TAL-900 (52.5 cm^2^), Zn-TAL-700 (12.5 cm^2^), and Zn-TAL-800 (10 cm^2^). The superior ECSA of Zn-TAL-1000 indicates greater exposure of active sites to the electrolyte, which can be attributed to its highly porous structure.

The rotating disk electrode technique was used to assess the ORR activity of the synthesized electrocatalyst materials and benchmark them against commercial Pt/C. As shown in Figure 6a, Zn-TAL-1000 demonstrated the highest onset potential (*E*_on_) of 0.98 V and a half-wave potential (*E*_1/2_) of 0.84 V, which is only 10 mV lower than that of Pt/C (0.85 V vs RHE). The Tafel slope values derived from the RDE data (Figure 6b) were similar to that of Pt/C (61 mV dec^–1^), indicating that Zn-TAL samples exhibit improved ORR kinetics.^65^ Additionally, Zn-TAL-1000 displayed a diffusion-limiting current density (*J*_L_) of 5.15 mA cm^–2^, further demonstrating its effective catalytic performance.

Figure 6c displays the RDE curves recorded for Zn-TAL-1000 at different rotation rates (ω) ranging from 620 to 2025 rpm, allowing for obtaining the Koutecky–Levich (K–L) plots (Figure 6d). The K–L plots at different potentials exhibit a strong linear relationship, indicating first-order ORR kinetics over the oxygen concentration in electrolyte across all electrocatalysts. The electron transfer number (*n*) for Zn-TAL-1000 was calculated to be around 3.5, close to the 4.0 value observed for Pt/C, suggesting that Zn-TAL-1000 predominantly follows a four-electron pathway during ORR. A comprehensive summary of all calculated kinetic parameters for each of the studied samples is provided in Table 3, with a comparative analysis of these parameters against Zn–N–C materials reported in the literature available in Supporting Information Table S5.

**Table 3:** Main Electrokinetic Parameters Obtained for Zn–N–C and Pt/C Samples

electrocatalyst	*E*_1/2_ (V vs RHE)	*E*_on_ (V vs RHE)	*n*	Tafel slope (mV dec^–1^)	*C*_dl_ (mF cm^–2^)	ECSA (cm^2^)
Zn-TAL-700	0.64	0.76	1.12	–64	0.5	12.5
Zn-TAL-800	0.73	0.82	2.37	–53	0.4	10
Zn-TAL-900	0.79	0.89	2.46	–51	2.1	52.5
Zn-TAL-1000	0.84	0.98	3.45	–53	2.3	57.5
Pt/C	0.85	0.98	4.00	–61	N/A	N/A

Based on the above discussion, it can be concluded that the promising ORR activity of Zn-TAL-1000 results from a combination of such factors as the incorporation of N-doped carbon and the presence of atomically dispersed Zn, which enhances the intrinsic reactivity for ORR. The hierarchical porosity, large surface area, and high pore volume of Zn-TAL-1000 material offer abundant accessible active sites and improve mass transport during the ORR.

The active site identification for ORR in Zn-TAL-derived catalysts is based on the synergistic roles of nitrogen species and Zn coordination. The XPS analysis indicates the presence of pyridinic, pyrrolic, and graphitic nitrogen in all pyrolyzed samples, which are well-recognized as active sites for ORR, particularly pyridinic nitrogen due to its ability to facilitate oxygen adsorption and electron transfer.^66−69^ Regarding the role of Zn, there is growing evidence that Zn can play an active role beyond being a structural template. Studies have shown that atomically dispersed Zn–N_*x*_ sites can contribute to ORR activity, exhibiting catalytic behavior comparable to Fe–N–C catalysts.^18,70^ For example, Zn–N_4_ coordination environments have been reported to facilitate oxygen adsorption while maintaining high durability, particularly in alkaline media.^70^ Furthermore, recent studies indicate that Zn single-atom sites, especially when coordinated with nitrogen and oxygen ligands (Zn–N_4_–O), can exhibit enhanced catalytic activity by optimizing the adsorption strength of *OH and reducing the energy barrier of the rate-determining step.^18^ In Zn-TAL-1000 catalyst, the evaporation of Zn at high temperatures suggests that Zn primarily acts as a template for forming the porous hollow structure. As the pyrolysis temperature increases, the specific surface area significantly enhances from 100 to 746 m^2^ g^–1^. This increase in porosity leads to a higher proportion of electrochemically active sites, further improving the ORR activity of the electrocatalysts. However, at lower pyrolysis temperatures, a portion of Zn may remain coordinated with nitrogen, contributing to the catalytic activity, as observed in Zn–N_*x*_-based SACs.^60^ The highest NO content in Zn-TAL-900 suggests the possibility of Zn existing in the form of ZnO or Zn–O–N coordination rather than Zn–N_*x*_, negatively influencing ORR activity.^71^

To assess the stability of Zn-TAL-1000, accelerated testing was conducted in alkaline media, involving 5000 cycles from 0.6 to 1.0 V vs RHE at a scan rate of 50 mV s^–1^ in an O_2_-saturated electrolyte. Post-stability polarization measurements (Figure 6f) confirmed that Zn-TAL-1000 retained significant catalytic activity for the ORR following extensive potential cycling, indicating resistance to electrochemical degradation under these test conditions. This aligns well with the theoretical study by Jin et al., which suggests that high binding energies exceeding the cohesive energy of Zn, along with the electron-withdrawing effect of N due to its higher electronegativity, reduce aggregation and stabilize Zn active sites in the structure.^29^

**Figure 7:**
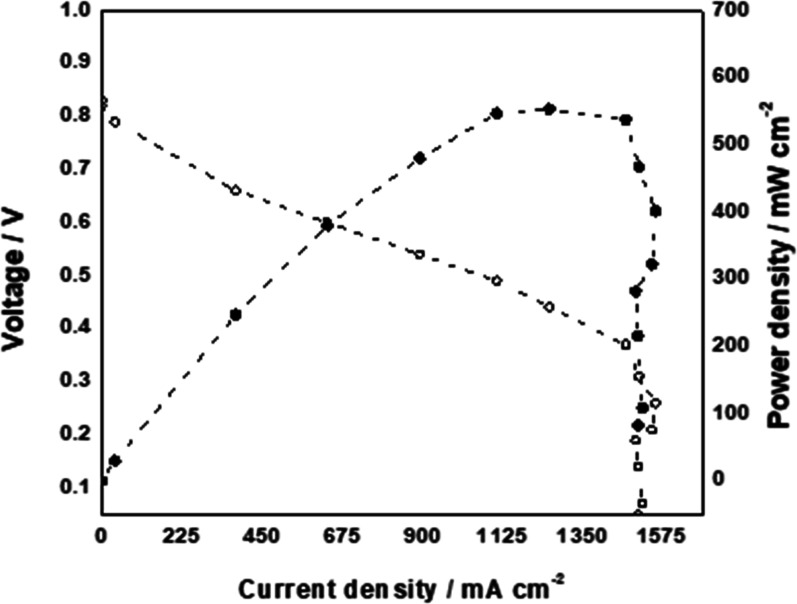
Polarization curve (empty symbols, Y1 axis) and power density curve (filled symbols, Y2 axis) of an H_2_–O_2_ AEMFC with Zn-TAL-1000 ORR cathode electrocatalyst. Test conditions: cathode and anode loadings of 1 mg_Zn/TAL-1000_ cm^–2^ and 0.6 mg_PtRu_ cm^–2^ and, respectively with an HDPE-based AEM. Cell temperature of 60 °C, cathode humidifier temperature of 56 °C and anode humidifier temperature of 54 °C and for O_2_ and H_2_, respectively with flow rates of 1 SLPM and back-pressure of 100 kPag for both electrodes.

The performance of the Zn-TAL-1000 cathode electrocatalyst was evaluated in an AEMFC operating at 60 °C, with the results illustrated in Figure 7. When paired with a PtRu/C anode, the AEMFC utilizing the Zn-TAL-1000 cathode achieved a peak power density of 553 mW cm^–2^ and a limiting current density of approximately 1500 mA cm^–2^. These results suggest that the Zn-TAL-1000 electrocatalyst may enhance the electrochemical performance of AEMFCs, indicating its potential relevance for further exploration in energy conversion applications.

## 4 Conclusions

In summary, a Zn–N–C electrocatalyst was successfully synthesized from the Zn-TAL MOF using an optimized pyrolysis process that preserved the hollow, porous structure essential for catalytic performance. This structure, verified by SEM and XRD analyses, remained stable at elevated temperatures without Zn aggregation. Electrochemical tests, including cyclic voltammetry and rotating disk electrode measurements, showed that Zn-TAL-1000 has a high onset potential (0.98 V) and a half-wave potential (*E*_1/2_) of 0.84 V, closely matching the performance of commercial Pt/C. Additionally, RDE results confirmed that Zn-TAL-1000 predominantly follows a four-electron ORR pathway, with an electron transfer number of 3.45. Accelerated stability testing demonstrated strong resistance to electrochemical degradation, with Zn-TAL-1000 maintaining significant ORR activity after 5000 cycles in alkaline media. Furthermore, in an anion-exchange membrane fuel cell, the Zn–N–C material pyrolyzed at 1000 °C exhibited a peak power density of 553 mW cm^–2^ at 60 °C. This work establishes Zn-TAL-derived Zn–N–C as a promising, cost-effective Pt-free electrocatalyst for fuel cell applications. Zn-TAL MOF is a versatile precursor for Zn–N–C electrocatalysts; however, it can also serve as a promising platform for developing advanced electrocatalysts. Its unique hollow sphere structure and rich carbon and nitrogen content enhance the incorporation of transition metal species, leading to improved performance in electrochemical devices such as metal-air batteries and fuel cells.

## Supporting Information Available

Detailed information regarding the physicochemical characterization of catalyst materials (SEM, TGA, XPS), additional electrochemical data, tables with SEM–EDX and XPS data, table of comparison of electrochemical performance with previously reported Zn–N–C catalysts (PDF)
