# Supplementary material for: Pyrolytic Transformation of Zn-TAL Metal–Organic Framework into Hollow Zn–N–C Spheres for Improved Oxygen Reduction Reaction Catalysis
Source: ACS Omega. 2025 Apr 12;10(15):15280–91. doi: 10.1021/acsomega.4c11318 (PMC12019727; doi:10.1021/acsomega.4c11318)
Supplement: Supplementary file 1 — ao4c11318_si_001.pdf [file ao4c11318_si_001.pdf]

## *Supporting Information*

*for*

**Pyrolytic Transformation of Zn-TAL MOF into Hollow Zn-N-C Spheres for Improved Oxygen Reduction Reaction Catalysis**

Gulnara Yusibova,[a] John C. Douglin,[b] Iuliia Vetik,[a] Jekaterina Pozdnjakova,[a] Kefeng Ping,[c] Jaan Aruväli,[d] Arvo Kikas,[e] Vambola Kisand,[e] Maike Käärik,[a] Jaan Leis,[a] Tiit Kaljuvee,[f] Peeter Paaver,[d] Sven Oras,[g] Łukasz Ciupiński,[h] Tomasz Plocinski,[h] Marina Konuhova,[i] Anatoli I. Popov,[i] Dario R. Dekel,[b,j] Vladislav Ivaništšev,[a] Nadezda Kongi[a*]

[a] *Institute of Chemistry, University of Tartu, Ravila 14a, 50411, Tartu, Estonia*
[b] *The Wolfson Department of Chemical Engineering, Technion – Israel Institute of Technology, Haifa 3200003, Israel*
[c] *Yichang Humanwell Pharmaceutical Co., Ltd, 19 Dalian Rd, Xiling District, Yichang 443005, Hubei, China*
[d] *Institute of Ecology and Earth Sciences, University of Tartu, Ravila 14a, 50411, Tartu, Estonia*
[e] *Institute of Physics, University of Tartu, Ostwaldi 1, 50411, Tartu, Estonia*
[f] *Department of Materials and Environmental Technology, Tallinn Technical University, Ehitajate tee 5, 19086 Tallinn, Estonia*
[g] *Institute of Technology, University of Tartu, Nooruse 1, 50411, Tartu, Estonia*
[h] *Warsaw University of Technology, Warsaw, Woloska 141, 02-507, Warsaw, Poland*
[i] *Institute of Solid State Physics, University of Latvia, 8 Kengaraga, LV-1063, Riga, Latvia*
[j] *The Nancy & Stephen Grand Technion Energy Program (GTEP), Technion – Israel Institute of Technology, Haifa 3200003, Israel*

* Corresponding author: nadezda.kongi@ut.ee (Nadezda Kongi)

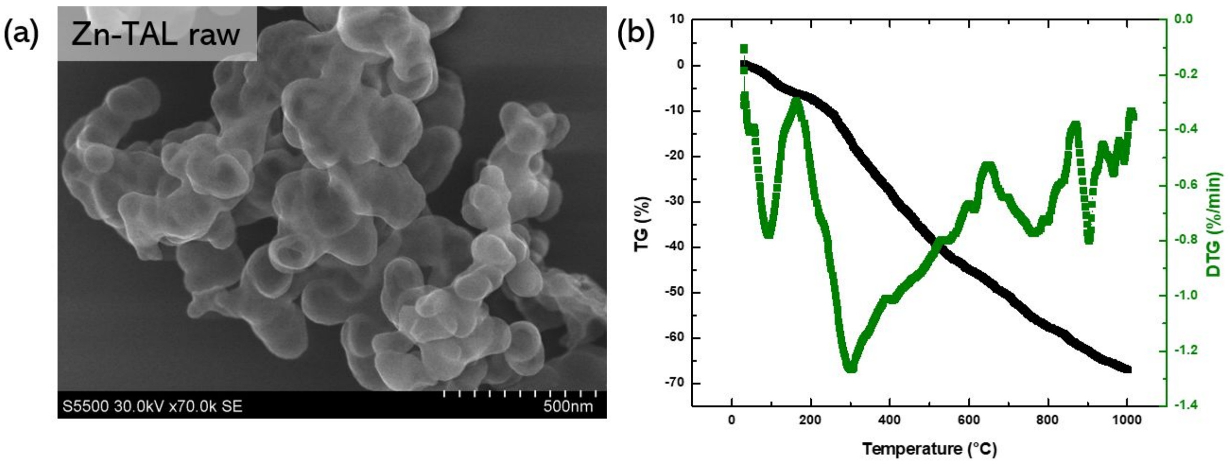


**Figure S1.** (a) Scanning electron microscopy image, and (b) TGA profile of Zn-TAL raw sample.

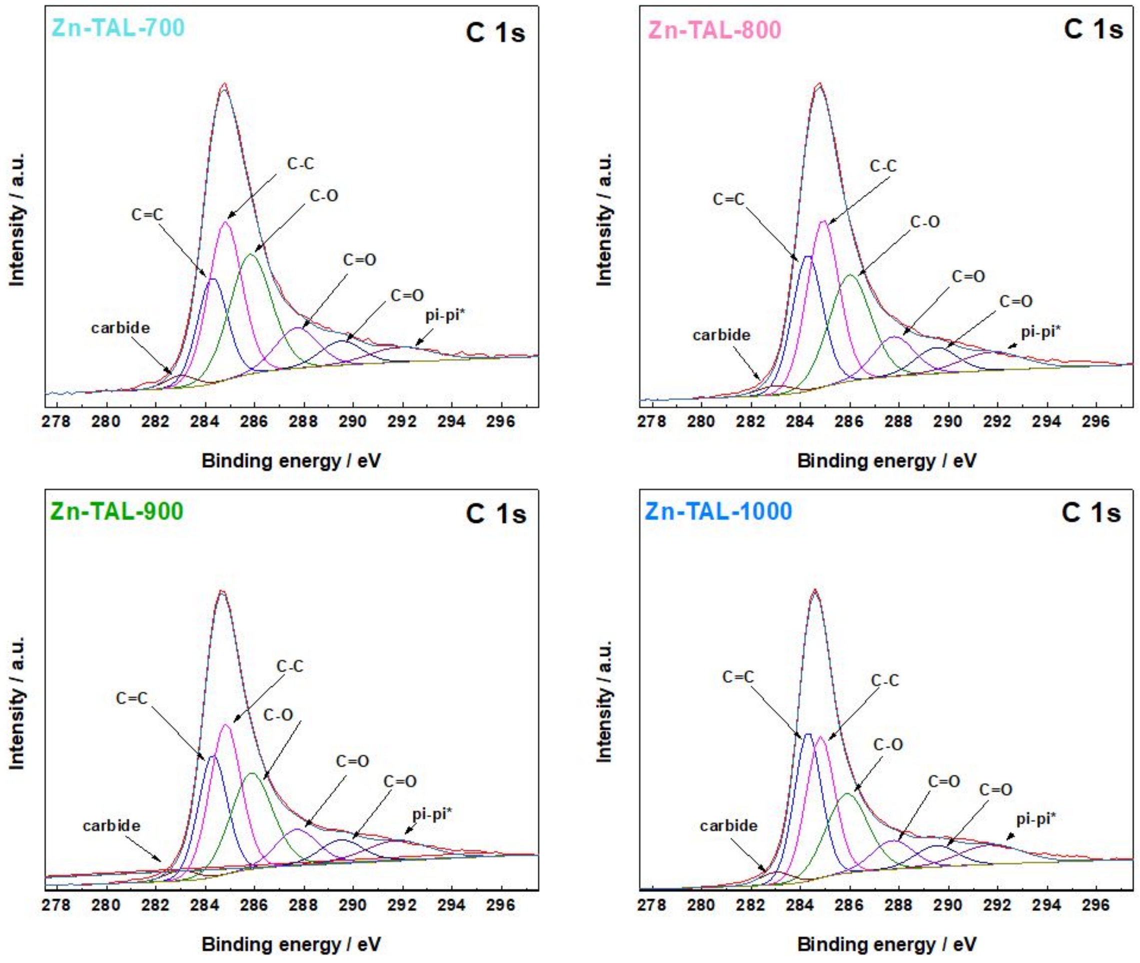


**Figure S2.** Deconvoluted C 1s XPS spectra for Zn-TAL derived catalysts materials.

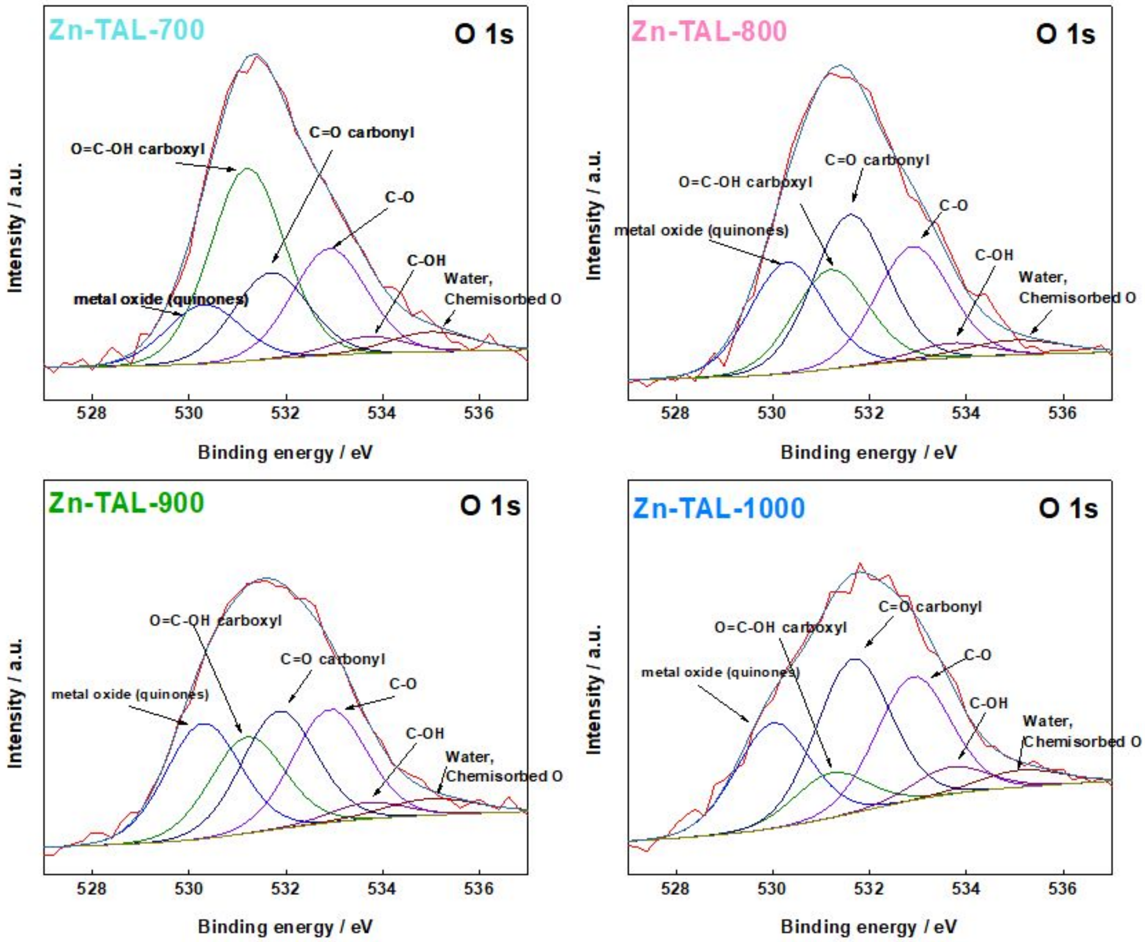


**Figure S3.** Deconvoluted O 1s XPS spectra for Zn-TAL derived catalysts materials.

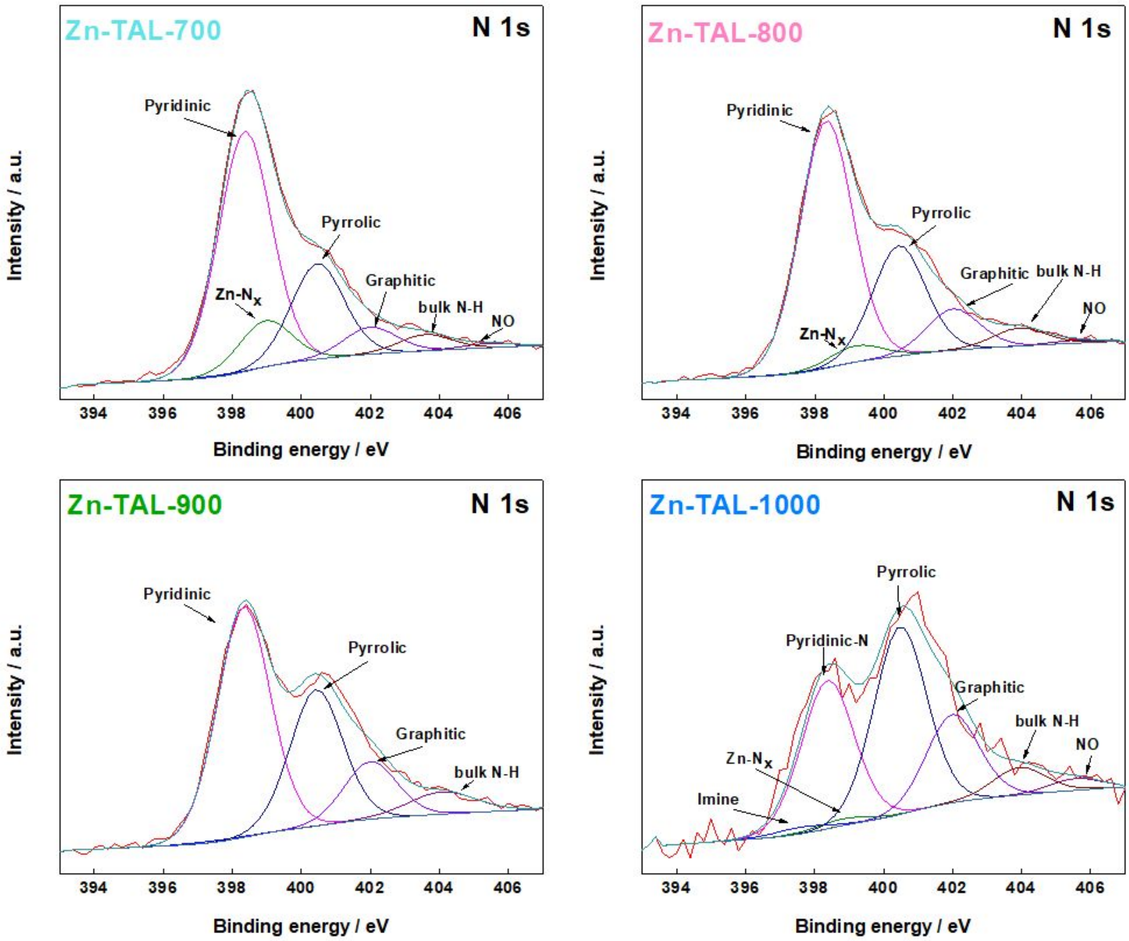


**Figure S4.** Deconvoluted N 1s XPS spectra for Zn-TAL derived catalysts materials.

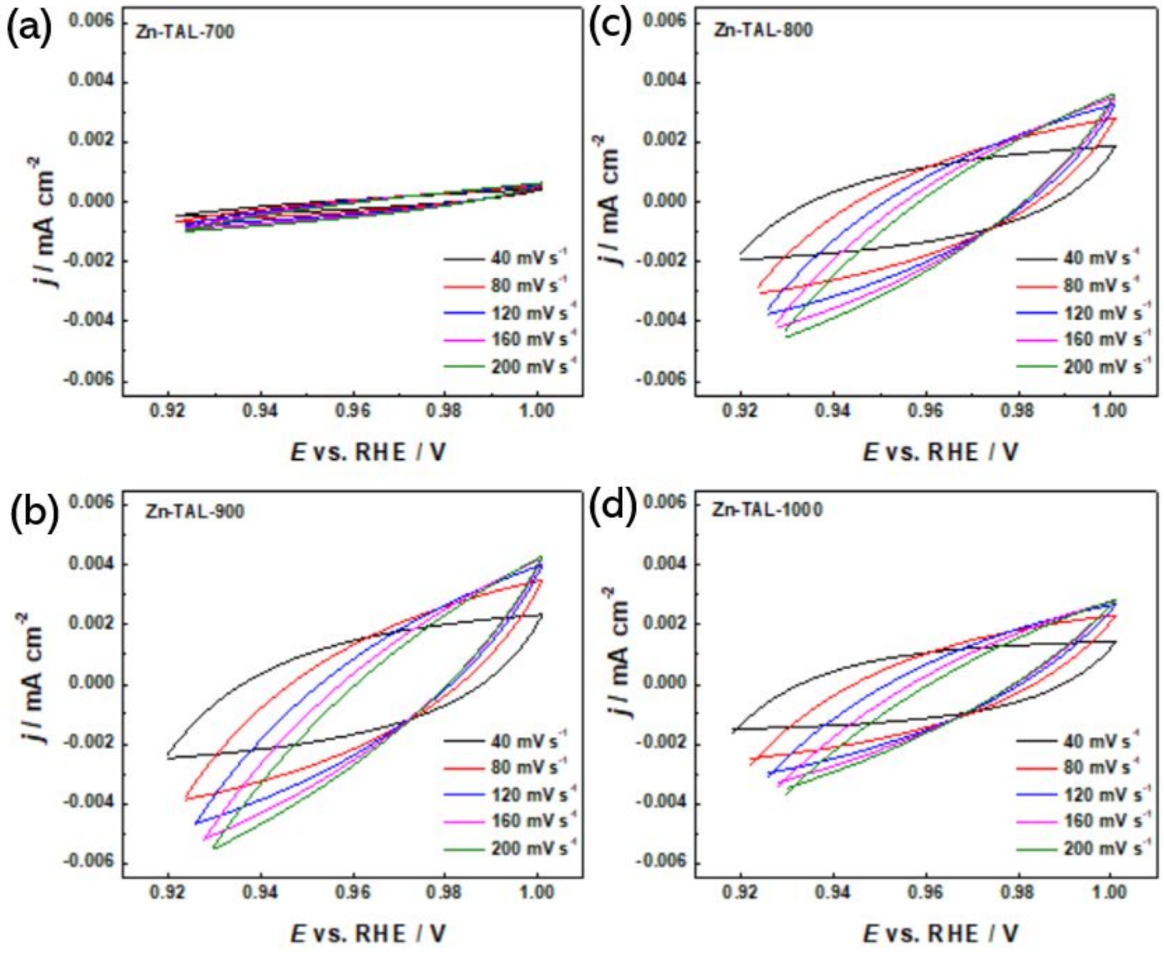


**Figure S5.** Non-Faradaic CV scans of (a) Zn-TAL-700, (b) Zn-TAL-800, (c) Zn-TAL-900, and (d) Zn-TAL-1000 at different scan rates in 0.1 M KOH.

**Table S1.** Elemental composition (at%) of Zn-TAL samples determined by SEM-EDX.

| Catalyst | C | N | Zn | O | Cl |
|---|---|---|---|---|---|
| **Zn-TAL raw** | 71.44± 0.36 | 17.30±0.17 | 3.87±0.03 | 7.20±0.07 | 0.19±0.02 |
| **Zn-TAL-700** | 82.30±0.46 | 9.79±0.16 | 3.41±0.03 | 3.66±0.06 | 0.84±0.03 |
| **Zn-TAL-800** | 86.41±0.40 | 11.67±0.14 | 1.71±0.02 | N/A | 0.21±0.02 |
| **Zn-TAL-900** | 94.11±0.43 | 4.57±0.10 | 1.02±0.01 | 0.30±0.02 | N/A |
| **Zn-TAL-1000** | 94.65±0.43 | 5.15±0.10 | 0.20±0.01 | N/A | N/A |

**Table S2.** Elemental composition (wt%) of Zn-TAL samples determined by SEM-EDX.

| **Catalyst** | **C** | **N** | **Zn** | **O** | **Cl** |
|---|---|---|---|---|---|
| **Zn-TAL raw** | 58.15± 0.30 | 16.42±0.16 | 17.17±0.12 | 7.80±0.07 | 0.45±0.05 |
| **Zn-TAL-700** | 68.80±0.38 | 9.55±0.16 | 15.51±0.14 | 4.07±0.06 | 2.07±0.09 |
| **Zn-TAL-800** | 78.59±0.36 | 12.38±0.15 | 8.47±0.08 | N/A | 0.57±0.04 |
| **Zn-TAL-900** | 89.29±0.41 | 5.06±0.11 | 5.27±0.07 | 0.37±0.03 | N/A |
| **Zn-TAL-1000** | 93.03±0.42 | 5.90±0.12 | 1.06±0.04 | N/A | N/A |

**Table S3.** Surface composition of C-groups (at.%) obtained from deconvoluted C 1s XPS spectra.

| **Catalyst** | sp2 | sp3 | C-O | C=O | pi-pi* | carbide |
|---|---|---|---|---|---|---|
| **Zn-TAL-700** | 12.35 | 20.8 | 19.59 | 10.61 | 3.73 | 1.44 |
| **Zn-TAL-800** | 17.43 | 22.64 | 18.85 | 11.41 | 4.93 | 1.4 |
| **Zn-TAL-900** | 18.58 | 24.89 | 21.19 | 12.76 | 6.37 | 1.75 |
| **Zn-TAL-1000** | 25.1 | 25.42 | 20.71 | 12.95 | 7.71 | 2.48 |

**Table S4.** Surface composition of N-groups (at.%) obtained from deconvoluted N 1s XPS spectra.

| **Catalyst** | Imine | Pyridinic | M-$N_x$ | Pyrrolic | Graphitic | NO | Bulk N-h |
|---|---|---|---|---|---|---|---|
| **Zn-TAL-700** | 0.04 | 8.58 | 1.66 | 3.41 | 0.96 | 0.16 | 0.58 |
| **Zn-TAL-800** | 0.03 | 7.25 | 0.48 | 3.24 | 1.21 | 0.08 | 0.53 |
| **Zn-TAL-900** | 0.03 | 4.25 | 0 | 2.51 | 1.07 | 0.52 | 0 |
| **Zn-TAL-1000** | 0.04 | 0.92 | 0.04 | 1.17 | 0.55 | 0.07 | 0.17 |

**Table S5.** Comparison of electrochemical performance in alkaline media of previously reported Zn-N-C catalysts.

| **Sample** | **$E_{1/2}$ (V vs. RHE)** | ***n*** | **Reference** |
|---|---|---|---|
| Zn-TAL-1000 | 0.84 | 3.45 | **This work** |
| Zn–N/S–C(S, Z) | 0.89 | 3.92 | 1 |
| Zn-N-C-2 | 0.85 | 3.5 | 2 |
| Zn-N4-O | 0.88 | 4 | 3 |
| A-Zn@NSG | 0.905 | 4 | 4 |
| Zn-N-C | 0.86 | 4.01 | 5 |
| Zn-N-C-1 | 0.873 | 4 | 6 |
| Zn/NC | 0.845 | 3.8 | 7 |
| Zn–SAs/UNCNS | 0.91 | 4 | 8 |

## References


(1) Liu, M.; Zhang, J.; Ye, G.; Peng, Y.; Guan, S. Zn/N/S Co-Doped Hierarchical Porous Carbon as a High-Efficiency Oxygen Reduction Catalyst in Zn–Air Batteries. *Dalton Trans.* **2023**, *52* (45), 16773–16779. https://doi.org/10.1039/D3DT03172A.

(2) Zhao, Z.; Xiong, Y.; Yu, S.; Fang, T.; Yi, K.; Yang, B.; Zhang, Y.; Yang, X.; Liu, X.; Jia, X. Single-Atom Zn with Nitrogen Defects on Biomimetic 3D Carbon Nanotubes for Bifunctional Oxygen Electrocatalysis. *Journal of Colloid and Interface Science* **2023**, *650*, 934–942. https://doi.org/10.1016/j.jcis.2023.06.182.

(3) Jin, Q.; Wang, C.; Guo, Y.; Xiao, Y.; Tan, X.; Chen, J.; He, W.; Li, Y.; Cui, H.; Wang, C. Axial Oxygen Ligands Regulating Electronic and Geometric Structure of Zn-N-C Sites to Boost Oxygen Reduction Reaction. *Advanced Science* **2023**, *10* (24), 2302152. https://doi.org/10.1002/advs.202302152.

(4) Jiang, R.; Chen, X.; Liu, W.; Wang, T.; Qi, D.; Zhi, Q.; Liu, W.; Li, W.; Wang, K.; Jiang, J. Atomic Zn Sites on N and S Codoped Biomass-Derived Graphene for a High-Efficiency Oxygen Reduction Reaction in Both Acidic and Alkaline Electrolytes. *ACS Appl. Energy Mater.* **2021**, *4* (3), 2481–2488. https://doi.org/10.1021/acsaem.0c03035.

(5) Hu, L.; Yu, F.; Wang, F.; Yang, S.; Peng, B.; Chen, L.; Wang, G.; Hou, J.; Dai, B.; Tian, Z.-Q. Overwhelming Electrochemical Oxygen Reduction Reaction of Zinc-Nitrogen-Carbon from Biomass Resource Chitosan *via* a Facile Carbon Bath Method. *Chinese Chemical Letters* **2020**, *31* (5), 1207–1212. https://doi.org/10.1016/j.cclet.2019.06.041.

(6) Li, J.; Chen, S.; Yang, N.; Deng, M.; Ibraheem, S.; Deng, J.; Li, J.; Li, L.; Wei, Z. Ultrahigh-Loading Zinc Single-Atom Catalyst for Highly Efficient Oxygen Reduction in Both Acidic and Alkaline Media. *Angewandte Chemie International Edition* **2019**, *58* (21), 7035–7039. https://doi.org/10.1002/anie.201902109.

(7) Wei, D.; Chen, L.; Tian, L.; Ramakrishna, S.; Ji, D. Zn Single Atoms/Clusters/Nanoparticles Embedded in the Hybrid Carbon Aerogels for High-Performance ORR Electrocatalysis. *Inorg. Chem.* **2023**, *62* (40), 16547–16553. https://doi.org/10.1021/acs.inorgchem.3c02417.

(8) Zhang, T.; Wang, F.; Yang, C.; Han, X.; Liang, C.; Zhang, Z.; Li, Y.; Han, A.; Liu, J.; Liu, B. Boosting ORR Performance by Single Atomic Divacancy Zn–N3C–C8 Sites on Ultrathin N-Doped Carbon Nanosheets. *Chem Catalysis* **2022**, *2* (4), 836–852. https://doi.org/10.1016/j.checat.2022.02.006.
